# Are the Analgesic Effects of Morphine Added to Transversus Abdominis Plane Block Systemic or Regional? A Randomized Clinical Trial

**DOI:** 10.1155/prm/9187270

**Published:** 2025-03-12

**Authors:** Meryem Onay, Osman Kaya, Elçin Telli, Ayten Bilir, Mehmet Sacit Güleç

**Affiliations:** ^1^Department of Anesthesiology and Reanimation, Faculty of Medicine, Eskisehir Osmangazi University, Eskisehir, Turkey; ^2^Department of Anesthesiology and Reanimation, Nizip State Hospital, Gaziantep, Turkey; ^3^Department of Gynecologic Oncology, Faculty of Medicine, Eskisehir Osmangazi University, Eskisehir, Turkey

**Keywords:** adjuvant, morphine, transversus abdominis plane block

## Abstract

**Background:** This study was designed to compare the effectiveness of the transversus abdominis plane (TAP) block with the addition of morphine to bupivacaine and the TAP block with bupivacaine plus intramuscular (IM) morphine. The aim of the study was to evaluate the effect of morphine administered with the TAP block on postoperative opioid consumption and pain scores and, secondarily, to determine whether the effect was systemic or local.

**Methods:** This prospective, double-blind, randomized controlled trial included 52 patients. In the IM group, morphine at a dose of 0.1 mg/kg based on ideal body weight (IBW) was administered IM. In addition, a bilateral TAP block was performed under ultrasound guidance using a total of 40 mL of 0.25% bupivacaine, with 20 mL injected on each side. In the TAP group, an ultrasound-guided TAP block, including a total of 40 mL of 0.25% bupivacaine and 0.1 mg/kg morphine according to the IBW of patients, was administered bilaterally.

**Results:** Total morphine consumption 24 h was 19.08 + 11.35 in the IM group and 11.81 + 7.02 in the TAP group, with an estimated difference in means of 7.2 (95% CI: 2.0, 12.5; *p*=0.008). The morphine consumption after 6, 12, and 24 h was lower in the TAP group than in the IM group (*p*=0.033, *p*=0.003, and *p*=0.008, respectively). The VAS scores at rest and during movement did not differ between the two groups. The total 24-h ondansetron consumption was higher in the IM group (*p*=0.046). The postoperative heart rates, blood pressure, and peripheral oxygen saturation at 0, 1, 6, 12, and 24 h did not differ significantly between the groups.

**Conclusions:** The addition of morphine to the TAP block may be an effective method for postoperative analgesia in gynecologic surgery and may not increase systemic side effects, due to the possible local effects of morphine administered interfacial.

**Trial Registration:** ClinicalTrials.gov identifier: NCT05420337

## 1. Introduction

Postoperative pain is associated with increased mortality and morbidity. Thus, effective management of postoperative pain is essential for minimizing postoperative complications, improving quality of life, and maximizing patient recovery [1]. A previous study reported that regional anesthetic techniques reduced postoperative opioid use, improved the quality of recovery, and enabled early mobilization in patients [2].

The transversus abdominis plane (TAP) block and its anatomical landmarks were first described by Rafi [3]. In 2007, an ultrasound-guided TAP block technique demonstrated a significant improvement in performance and success rate. Notably, TAP blocks prevent pain in the anterolateral wall of the abdomen, which is innervated by the anterior and lateral cutaneous branches of the thoracolumbar nerves (T6–L1). The block is performed by applying local anesthetic agents to the fascia between the internal oblique and transverse abdominis muscles. It is used as a component of multimodal analgesia in lower abdominal surgery, owing to its safety and effectiveness in gynecologic, urologic, and pediatric cases [4–6].

Opioids are widely used systemically; however, their use is limited due to side effects and potential delays in postoperative recovery [7]. Opioid receptors are found in both the central and peripheral nervous systems, as well as in nonneuronal tissues [8]. Human and animal studies have shown that inflammation increases the synthesis of opioid receptors in the dorsal root ganglion neurons and enhances the accumulation and transport of opioid receptors in the peripheral terminals of sensory neurons [8]. This has led to new approaches for the development of effective peripheral analgesic techniques.

Our primary aim in this study was to compare the effects of TAP block with bupivacaine plus morphine versus TAP block with bupivacaine plus intramuscular (IM) morphine on postoperative pain scores and total opioid consumption in lower abdominal surgery. Secondarily, we aimed to investigate whether the addition of morphine to the TAP block exerted a systemic or local effect.

## 2. Methods

This randomized controlled, double-blinded study was approved by the Ethics Committee (approval no. 2021/23). This study was conducted between August 2022 and March 2023. All patients provided verbal and written informed consent to undergo the block procedures and for the maintenance of study records. The trial included 52 patients between the ages of 18 and 65 who underwent gynecological surgery with American Society of Anesthesiologists (ASA) physical status classifications I–II. Exclusion criteria for this study included patients with known allergies to the study drugs, significant cardiac, respiratory, renal, or hepatic diseases, bleeding diathesis, or psychiatric conditions that could impact pain perception and assessment.

### 2.1. General Anesthesia Procedure

Following routine monitoring, a standard general anesthesia protocol was administered to patients upon admission to the operating room. General anesthesia was induced using propofol at a dosage of 2-3 mg/kg, rocuronium at 0.6 mg/kg, and remifentanil at 0.1–0.3 mcg/kg. Following intubation, anesthesia was maintained using a mixture of 2%–3% sevoflurane. The remifentanil infusion was titrated to a dose of 0.1–0.25 mcg/kg/min to ensure heart rate and blood pressure remained within 20% of baseline value. The patients underwent gynecological surgery (unilateral or bilateral salpingo-oophorectomy, kistectomy, total abdominal hysterectomy, and cancer staging surgery) classified as simple, intermediate, or staging surgery. Laparotomy was accompanied by a low midline vertical incision or high midline vertical incision in the supine position. Paracetamol (1 g) was administered to all patients 30 min before the end of the operation. Demographic data such as age, body mass index (BMI), ideal body weight (IBW), operation type, duration, and complications were recorded.

At the end of the surgery, patients were randomly assigned into two groups: the IM groupand the TAP group. For randomization, a computer-generated table of random numbers was utilized, and the sealed envelope method was employed. A TAP block was administered to all patients in both groups while in the supine position under general anesthesia. The blocks were performed by the same anesthesiologist (M.O.), who was not involved in data collection.

The TAP block was initiated after cleaning the skin with a 10% povidone–iodine antiseptic solution. It was performed using a USG linear probe (Samsung HS50, Seoul, South Korea) at the end of the surgery. The internal oblique and transversus abdominis muscles were identified at the midaxillary line between the costal margin and the iliac crest, and a 21-gauge, 85-mm needle (Vygon [UK] Ltd, Swindon, United Kingdom) was advanced using the in-plane technique.

### 2.2. Group IM Procedure

Morphine was administered at 0.1 mg/kg IBW intramuscularly, and a TAP block was applied at the end of the operation. After confirming the location with 1-2 mL of saline, a total of 40 mL of 0.25% bupivacaine was administered bilaterally, with 20 mL injected on each side.

### 2.3. Group TAP Procedure

After hydrodissection and confirmation of the location of the TAP block using 1-2 mL of saline, a total of 40 mL of 0.25% bupivacaine + 0.1 mg/kg morphine according to the IBW of patients was administered bilaterally.

### 2.4. Postoperative Follow-Up

After the procedure, neuromuscular blockade was reversed using sugammadex at a dose of 2 mg/kg. Patients with a modified Aldrete score of 9 or higher were transferred from the postoperative anesthesia unit. Each patient was provided with an intravenous morphine patient-controlled analgesia (PCA) device. The PCA solution had a morphine concentration of 0.5 mg/mL, and a bolus dose of 1 mg was administered with a 10-min lock-out period and no basal infusion. In addition, 1 g of paracetamol was given intravenously every 8 h. Postoperatively, another researcher, who was blinded to the patient groups, conducted evaluations at 1, 6, 12, and 24 h. They recorded visual pain scores (using visual analog scale [VAS]) at rest and during movement, hemodynamic values, morphine consumption, nausea and vomiting scores, itching, Ramsay sedation scale scores, and any postoperative complications.

Patients were evaluated for postoperative pain both at rest and during movement. Their pain levels were measured using a VAS, with separate scores for rest (VAS_Rest_) and movement (VAS_Movement_). The scale ranged from 0 to 10 cm, where 0 indicated *no pain* and 10 indicated *severe pain*. VAS_movement_ evaluated the severity of pain with patient coughing. Patients with postoperative pain VAS scores of > 4 were administered diclofenac sodium IM as a rescue analgesic. In addition, a 4-point scale (1 = *none*; 2 = *minor*; 3 = *moderate*; 4 = *severe*) was used to assess the severity of nausea, vomiting, and itching. Patients with a nausea and vomiting score greater than 2 were administered 8 mg of ondansetron. The sedation level was evaluated using the Ramsay sedation scale.

### 2.5. Sample Size Estimation

The sample size of the present study was calculated using G∗Power 3.1.9 (G∗Power, Universität Düsseldorf, Germany). Based on the study [4], the primary aim was to compare the results of 24-h total opioid consumption in two groups; notably, the Type I error was *α* = 0.05, the effect size was high (0.80), the targeted power of the test was 1 − *β* = 0.80, and the sample size required for statistical analyses was determined to be 26 for each group (total = 52).

### 2.6. Statistical Analysis

The normality of the distribution of the continuous variables was evaluated using the Shapiro–Wilk normality test. Student's *t*-test and repeated-measures analysis of variance were used for intergroup comparisons of continuous variables. Sidak multiple comparison tests were performed using repeated-measures analysis of variance. Pearson's, Yates, and Fisher's exact tests and Monte Carlo exact chi-square analyses were used to compare categorical variables between the groups. The level of significance was set at *p* < 0.05. All analyses were performed using IBM SPSS Statistics Version 25.

## 3. Results

**Figure 1:**
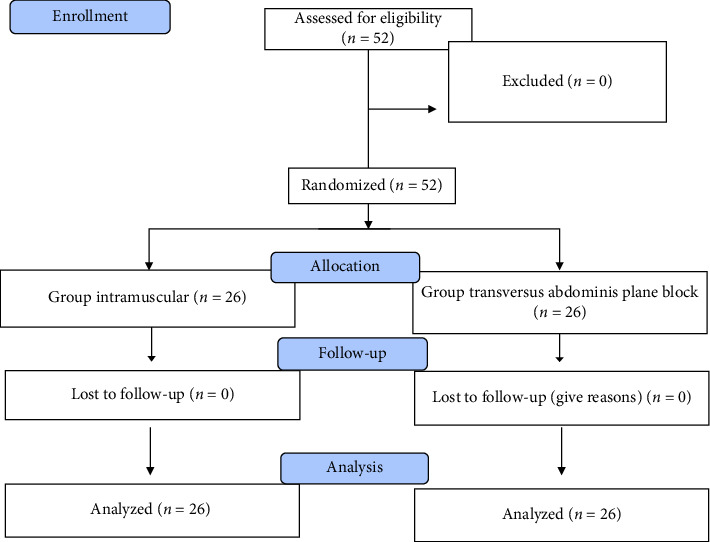
Consort flow diagram.

In this study, 52 patients were included (Figure 1). There were no significant differences between the groups in terms of demographic data such as age, ASA class, BMI, IBW, operation type, and operation time (Table 1).

The TAP group showed no significant difference in VAS scores at rest and during movement compared to the IM group (Tables 2 and 3).

Total 24-h morphine consumption was lower in the TAP group (IM group: 19.08 ± 11.35 and TAP group: 11.81 ± 7.02, *p* : 0.008). Specifically, morphine consumption at the 6^th^, 12^th^, and 24^th^ hours was significantly lower in the TAP group than in the IM group (*p*=0.033, *p*=0.003, and *p*=0.008, respectively) (Table 4). The levels of morphine demand on postoperative hours 1 and 6 were similar in both groups, while those on postoperative hours 12 and 24 were significantly higher in the IM group and differed among groups (*p*=0.936, *p*=0.109, *p*=0.032, and *p*=0.036, respectively). Rescue analgesia was not statistically significant between the two groups (*p*=0.200).

The number of patients with nausea (in the first hour: Group IM, score 1 = % 42.3, 2 = %19.2, 3 = %34.6, and 4 = % 3.8; Group TAP, score 1 = % 50, 2 = % 30.8, 3 = % 19.2, and 4 = % 0) and vomiting (Group IM, score 1 = % 88.5, 2 = % 7.7, 3 = % 3.8, and 4 = % 0; Group TAP, score 1 = % 92.3, 2 = % 3.8, 3 = % 3.8, and 4 = % 0) and itching and Ramsey sedation scale scores were similar between the groups at the first hour. However, the total 24-h ondansetron consumption was significantly higher in the IM group (*n* = 14 [% 53.8]) than in the TAP group (*n* = 6 [%23.1]) (*p*=0.046). Postoperative ondansetron was only administered in the first hour with a score > 2, and it was not needed in the other hours.

The heart rate, blood pressure (systolic, diastolic, and mean blood pressure), and peripheral oxygen saturation (*p*=0.609, *p*=0.617*p*=0.492, *p*=0.462, and *p*=0.765, respectively) at 0, 1, 6, 12, and 24 h postoperatively were not significantly different between the groups. A peripheral oxygen saturation of < 90 was observed at the 6^th^ hour postoperatively in one patient in the IM group and at the 24^th^ hour in one patient in the TAP group.

## 4. Discussion

In the present study, we evaluated the effects of morphine as an adjuvant to the TAP block, which has been proven to be effective for postoperative analgesia in patients undergoing gynecologic surgery under general anesthesia, and compared those with the IM morphine plus TAP block. The addition of morphine to the TAP block decreased the total use of opioids without increasing systemic side effects, such as nausea, vomiting, pruritus, hypotension, respiratory depression, and sedation, compared with systemic administration of the same dose of morphine. This suggests that interfacial morphine has a local effect besides its systemic effect.

Peripheral nerve blocks with local anesthetics are widely used in extremity surgeries to increase the analgesic effect and to reduce opioid use, length of hospital stay, and hospital costs [9]. The duration of analgesia can be prolonged by increasing the dose of local anesthetic; however, this may increase neurotoxicity and potential side effects [9]. In particular, in single-shot peripheral blocks, various adjuvants (opioids, epinephrine, magnesium, and dexmedetomidine) are administered along with local anesthetics to prolong the duration of analgesia [9, 10]. Adjuvants exert this effect through various mechanisms such as local vasoconstriction affecting systemic absorption, direct effects on peripheral nerves, and systemic anti-inflammatory effects [9].

To the best of our knowledge, there is no clear opinion regarding the addition of opioids to local anesthesia in peripheral nerve blocks [11]. It has been reported that the addition of morphine to the axillary brachial plexus block improves the quality of the block and contributes to postoperative recovery [11, 12]. However, some studies have suggested that the addition of morphine to intercostal and interscalene blocks does not prolong the duration of analgesia of these blocks, and perineural morphine does not surpass the benefits of intravenous and IM morphine. The side effects of morphine have been reported to be more predominant in such cases [10, 13, 14]^.^

Opioids are potential analgesics that exert pharmacological effects by binding to and activating specific nerve receptors [8]. A growing number of experimental studies in the relevant literature have suggested that peripheral opioid receptors are activated by inflammatory tissue changes and that endogenous opioids modulate the inflammatory process. Some studies suggest that local administration of exogenous opioid agonists exerts an analgesic effect by activating peripheral opioid receptors in the inflamed tissue [8, 15]. Analgesic activity occurs without the activation of the opioid receptor in the central nervous system; therefore, side effects such as respiratory depression, nausea, impaired consciousness, and addiction are thought to be unrelated to peripheral opioid activity [8, 16].

The TAP block is widely used for postoperative analgesia in abdominal surgery [17]. The addition of dexamethasone and magnesium to the TAP block is reportedly associated with lower pain scores [1, 17–19]. In patients undergoing cesarean section, the use of a TAP block, in addition to the administration of 4-mg dexamethasone on each side, has been known to prolong the duration of analgesia, reduce opioid consumption, and provide beneficial antiemetic effects, which further provide better postoperative recovery and maternal satisfaction [18–20]. However, a previous study reported that the use of 50-mcg fentanyl as an adjuvant in cesarean section did not contribute to the quality and duration of analgesia in the TAP block [21]. In gynecologic surgeries, the addition of 1 mcg/kg dexmedetomidine and 200-mg magnesium to the TAP block demonstrated better analgesia, prolonged the duration of analgesia, lowered analgesia consumption, and improved patient recovery [7, 22].

To the best of our knowledge, the study by El Sherif, Mohamed, and Kamal is the only randomized controlled trial to evaluate the effects of morphine addition to the TAP block. They compared the outcomes of using a TAP block along with 10 mg of morphine sulfate added to the local anesthetic and using a TAP block with a local anesthetic alone in lower abdominal cancer surgery. They reported that the combination decreased morphine consumption (GB: 10.70 ± 3.09 and GM: 5.33 ± 1.28) without increasing side effects [4]. However, in their study, morphine was added to the TAP block administered after the induction of anesthesia and was not added to the total dose. This suggests that the systemic effects of morphine added to the TAP block were neglected in their study. In the present study, morphine doses were determined according to the IBW of the patients and administered in addition to the TAP block. Although a lower dose was administered, our study reported results similar to those of a previous study, indicating that the addition of morphine to the TAP block decreased the total morphine consumption.

In a study by Riberro, Joel, and Zeppetella, who evaluated the bioavailability of 10 mg of morphine gel applied to skin ulcers, morphine and its metabolites were not detected systemically, indicating that the effect of morphine was local [16]. However, in a literature review on intra-articular morphine administration, it was reported that morphine provided analgesia for ≤ 24 h, its effect was possibly dose-dependent, and its systemic effect could not be ignored [23]. In that study, systemic effects, such as nausea, vomiting, pruritus, sedation, hypotension, and respiratory depression, were statistically similar; however, these effects were greater during systemic administration.

Morphine is a long-acting synthetic opioid with a late onset of action due to its low lipophilicity [24]. In this study, morphine was preferred as the adjuvant because of its low cost, accessibility, and long-acting effects. It was administered intramuscularly and fascially at equal doses to evaluate whether the effects were systemic or local. In the relevant literature, morphine doses administered to patients ranged from 10 mg to 0.1 mg/kg [4, 12, 15, 16, 25]. However, considering the systemic administration to the control group, the morphine dose was determined to be 0.1 mg/kg IBW. Although nausea and vomiting scores ≥ 2 were similar between the groups at the first hour, the number of patients who were administered ondansetron owing to nausea and vomiting scores > 2 was higher in the IM group. Therefore, it can be said that the effect of morphine in the facial plane starts later and its primary effect is local.

In the study of El Sherif et al., it was seen that adding 10-mg morphine to bupivacaine in serratus anterior plane block in patients who underwent modified radical mastectomy improved postoperative analgesia. It was observed that this effect was attributed merely to local mechanisms of action [26]. Similarly, Latzke et al. [27] stated in their study that the effect of local anesthetic applied in the TAP block is topical, but it varies according to localization.

The anterolateral abdominal wall is innervated by thoracolumbar nerves that become intercostal, subcostal, ilioinguinal/iliohypogastric nerves. These branches are adjacent to the deep circumflex iliac artery and the deep inferior epigastric artery. The most important concerns at this location are the vascularity of the TAP and the possibility of systemic toxicity caused by the high volume of local anesthetic administered [28]. In this case, it may lead to the speculation that the dose of local anesthesia and the need for repeated TAP block with a catheter can be reduced when morphine is added. However, further studies are needed to confirm this hypothesis.

This study had some limitations. First, the sample size was determined based on postoperative opioid consumption, not complications. The number of patients was insufficient to assess the systemic effects. Second, serum morphine levels were not studied to assess the systemic effects of morphine, which were clinically evaluated. Finally, the TAP block was recommended as part of multimodal analgesia; therefore, the inclusion of a placebo group of subjects who did not undergo the block was not planned in this study.

In conclusion, adjuvant morphine administered together with the TAP block as a multimodal analgesia component for postoperative analgesia in patients undergoing gynecological surgery creates analgesia in the postoperative period, due to the possible local effects of morphine administered interfacial, compared to systemic administered.

## Figures and Tables

**Table 1:** Demographic and clinical data.

	Group IM (*n* = 26)	Group TAP (*n* = 26)	*p* value
Age (years)	48.46 ± 11.21	48.0 ± 11.25	0.883
Body mass index (BMI)	27.73 ± 3,91	27.31 ± 4.97	0.735
Ideal body weight (IBW)	54.08 ± 4.62	53.85 ± 5.23	0.867
ASA I/II	10/16	8/18	0.771
Surgical incision	18/8	16/10	0.771
Low midline vertical incision/high midline vertical incision			
Type of surgery simple/medium/staging	4/12/10	8/9/9	0.404
Operative time (minutes)	147.19 ± 47.64	137.62 ± 62.35	0.537

*Note:* Data are expressed as mean ± standard deviation or number. ASA = American Society of Anesthesiologists physical status scores.

Abbreviations: Group IM = group intramuscular, Group TAP = group transversus abdominis plane block.

**Table 2:** Assessment of visual analog scale values of the groups at rest.

Time	Group IM (*n* = 26)	Group TAP (*n* = 26)	*p* value	Difference in means, 95% CI
Hour 1	3.35 ± 1.09	3.38 ± 0.94	0.892	−0.03 (−0.6–0.5)
Hour 6	2.50 ± 1.44	2.38 ± 1.35	0.768	0.1 (−0.6–0.8)
Hour 12	1.96 ± 1.14	1.88 ± 1.36	0.827	0.07 (−0.6–0.7)
Hour 24	1.81 ± 1.38	1.88 ± 1.33	0.839	−0.07 (−0.6–0.8)

*Note:* Visual analog scale values of the groups at rest is expressed as mean ± SD.

Abbreviations: Group IM = group intramuscular, Group TAP = group transversus abdominis plane block.

**Table 3:** Assessment of visual analog scale values of the groups during movement.

Time	Group IM (*n* = 26)	Group TAP (*n* = 26)	*p* value	Difference in means, 95% CI
Hour 1	4.77 ± 1.45	4.35 ± 0.79	0.198	0.4 (−0.2–1.0)
Hour 6	3.77 ± 1.75	3.81 ± 1.60	0.934	−0.03 (−0.9–0.8)
Hour 12	3.23 ± 1.58	3.15 ± 1.71	0.867	0.07 (−0.8–0.9)
Hour 24	3.58 ± 1.88	4.50 ± 1.92	0.086	−0.9 (−1.9 to 0.1)

*Note:* Visual analog scale values of the groups during movement is expressed as mean ± SD.

Abbreviations: Group IM = group intramuscular, Group TAP = group transversus abdominis plane block.

**Table 4:** Assessment of postoperative morphine consumption.

Time	Group IM (*n* = 26)	Group TAP (*n* = 26)	*p* value	Difference in means, 95% CI
Hour 1 (mg)	3.35 + 1.05	3.35 + 1.12	1.000	0.0 (−0.6–0.6)
Hour 6 (mg)	8.31 + 4.41	5.85 + 3.66	0.033^∗^	2.4 (0.2–4.7)
Hour 12 (mg)	12.00 + 6.29	7.38 + 3.99	0.003^∗^	4.6 (1.6–7.5)
Hour 24 (mg)	19.08 + 11.35	11.81 + 7.02	0.008^∗^	7.2 (2.0–12.5)

*Note:* Postoperative morphine consumption (mg) is expressed as mean ± SD.

Abbreviations: Group IM = group intramuscular, Group TAP = group transversus abdominis plane block.

⁣^∗^*p* < 0.05 indicates statistically significant values.

## Data Availability

The datasets generated during and/or analyzed during the current study are not publicly available due to restrictions of the local ethical committee but are available from the corresponding author on reasonable request.
